# Graphene-Based Nanoscale Vacuum Channel Transistor

**DOI:** 10.1186/s11671-018-2736-6

**Published:** 2018-10-04

**Authors:** Ji Xu, Zhuyan Gu, Wenxin Yang, Qilong Wang, Xiaobing Zhang

**Affiliations:** Joint International Research Laboratory of Information Display and Visualization, School of Electronic Science and Engineering, Southeast University, Nanjing, 210096 China

**Keywords:** Nanoscale vacuum channel, Graphene, In situ measurement

## Abstract

We report the fabrication and electrical performance of nanoscale vacuum channel transistor (NVCT) based on graphene. Ninety-nanometer-width vacuum nano-channel could be precisely fabricated with standard electron beam lithography process. The optimization and treatment of surface damage and adhesive residue on graphene are carried out by ultrasonic cleaning and thermal annealing. Additionally, in situ electric characteristics are directly performed inside a vacuum chamber of scanning electron microscope (SEM) with the nanomanipulator. By modulating the gate voltage, the NVCT could be switched from off-state to on-state, exhibiting an on/off current ratio up to 10^2^ with low working voltages (< 20 V) and leakage current (< 0.5 nA). Furthermore, the nanoscale vacuum channel could enable to scale down the size of vacuum devices with high integration, making NVCT a promising candidate for high speed applications.

**Electronic supplementary material:**

The online version of this article (10.1186/s11671-018-2736-6) contains supplementary material, which is available to authorized users.

## Background

As the traditional Si-based technology gradually reaches the minimize limitation, many efforts have been made in the novel nanostructures or low-dimensional materials [1–7]. Among these prominent issues, transistors composed of nanoscale vacuum channels or known as the nanogap have been steadily attracting attentions [8–10]. Distinct from the early vacuum tubes with high-power consumption and difficulty for high integration, the nanogap structures are more prospective for the modern nanoelectronics. For conventional field effect transistors (FETs), the carriers may collide with the optical and acoustic phonons during the transport. Also, intrinsic graphene-based FETs were found to have an on–off current ratio less than 10 due to the lack of a bandgap, which are not suitable for modern integrated logic circuits. Intrinsically, electrons could ballistically travel through the nanoscale vacuum channel while suffering from collision or scattering in the semiconductors. And the vacuum nano-devices could be compatible with standard silicon process and combine the advantages of ballistic transport with miniaturization and integration. Thus, the nanoscale vacuum channel transistors (NVCTs) may output high frequency [9, 11], on/off ratio [12], or fast temporal response [13] with low working voltage. More importantly, the NVCT is proved to retain the advantages of the traditional vacuum tubes that operate normally in the extreme conditions, like exposure of ionizing radiation or high temperature [8]. The development of manufacturing technology can open up enormous opportunities for creating nanoscale vacuum channel, which might be compatible with modern integrated circuit (IC).

As a result, many attempts have been made to downscale the vacuum channel into nanogap and construct three terminal junctions. For instance, the vertical structure was widely utilized in the traditional vacuum electronic devices [14, 15]. Researchers have proposed different types of vertical NVCTs, where the electrons could emit directly out of plane, e.g., the slit-type vacuum transistor [16], or the Spindt-type NVCT [17]. However, the vertical structure could hardly be compatible with CMOS process. Compared with up–down structure, the planar NVCT are more prospective for future integration as the nanogap is variable with mask layout, including electron beam lithography (EBL) [18], focused ion beam (FIB) [19], or nanoimprinting [20]. Recently, planar-type vacuum transistors with nanogap channel have been fabricated with traditional semiconductor processing. Meyyappan et al. demonstrated a back-gate vacuum nano-channel transistor with standard silicon semiconductor processing, showing high-frequency switching characteristics with negligible leakage current [9]. In order to enhance the gate controllability, they further fabricated a surround-gate NVCT consists of sub-50-nm vacuum channel, and the device was proven to stand against ionizing radiation (proton and Gamma ray) and high temperature (200 °C) [8]. Wei et al. successfully fabricated a graphene-based vacuum transistor with better electrical performance than those graphene-based solid-state transistors. With superior on/off current ratio and low working voltages, the graphene NVCT are expected to be applied in severe environments such as electromagnetic radiation or extreme temperature [12]. Our previous work also precisely fabricated sub-30 nm aligned nanogap arrays with a well-controlled process [21]. The experimental results above indicate that the vacuum nano-devices, composed of the nanoscale vacuum channel, have the advantages of high response speed, low operating voltage, and superior switching performance and, more importantly, could be compatible with standard silicon process and combine the advantages of ballistic transport with miniaturization and integration. In particular, the nano-channel that smaller than the mean free path of electron can behave as vacuum without scattering or collision. Thus, the NVCT may function in low vacuum environment or even atmosphere, paving the way for a new generation of high performance, high-speed and low-cost vacuum electronic devices.

Here, we report on the fabrication of a graphene-based NVCT using optimized wet transfer method and standard EBL processing. Vacuum nano-channel of 90 nm has been achieved with a back-gate structure, which could modulate the electric field of emitting area and the electron transmission through emitter to collector. In situ electric characteristics are performed in the vacuum chamber of scanning electron microscope (SEM) with a nanomanipulator, showing the basic functionality with high on/off current ratio, low work voltage, and leakage current. Importantly, we believe that further downscaling of the channel size could fulfill high speed, high reliability, and low-cost applications for modern electronics.

## Methods

### Wet Transfer

In this report, large-scale graphene was directly grown on the Cu foil by thermal chemical vapor deposition (CVD) at 1020 °C with CH_4_ (20 sccm) and H_2_ (40 sccm) [22]. Among various transfer techniques for CVD-grown graphene, the mainstream method is the chemical transfer using PMMA as a support layer. Firstly, a PMMA layer was spin-coated on the graphene/Cu film and baked at 100 °C for 5 min to solidify PMMA. After etching in the FeCl_3_:HCl:H_2_O solution (molar mass ratio of 1:1:1) for 90 min, the remaining PMMA/graphene film was transferred and soaked in the deionized water for 5 min. This cleaning operation was repeated four or five times to fully remove the etching solution residue. Then, the PMMA/graphene film was transferred to the SiO_2_/Si substrates and dried at 100 °C for 5 min, removing the residual water between the membrane and substrate. Lastly, the sample was soaked in the acetone solution for an hour to remove the PMMA support layer.

**Fig. 1:**
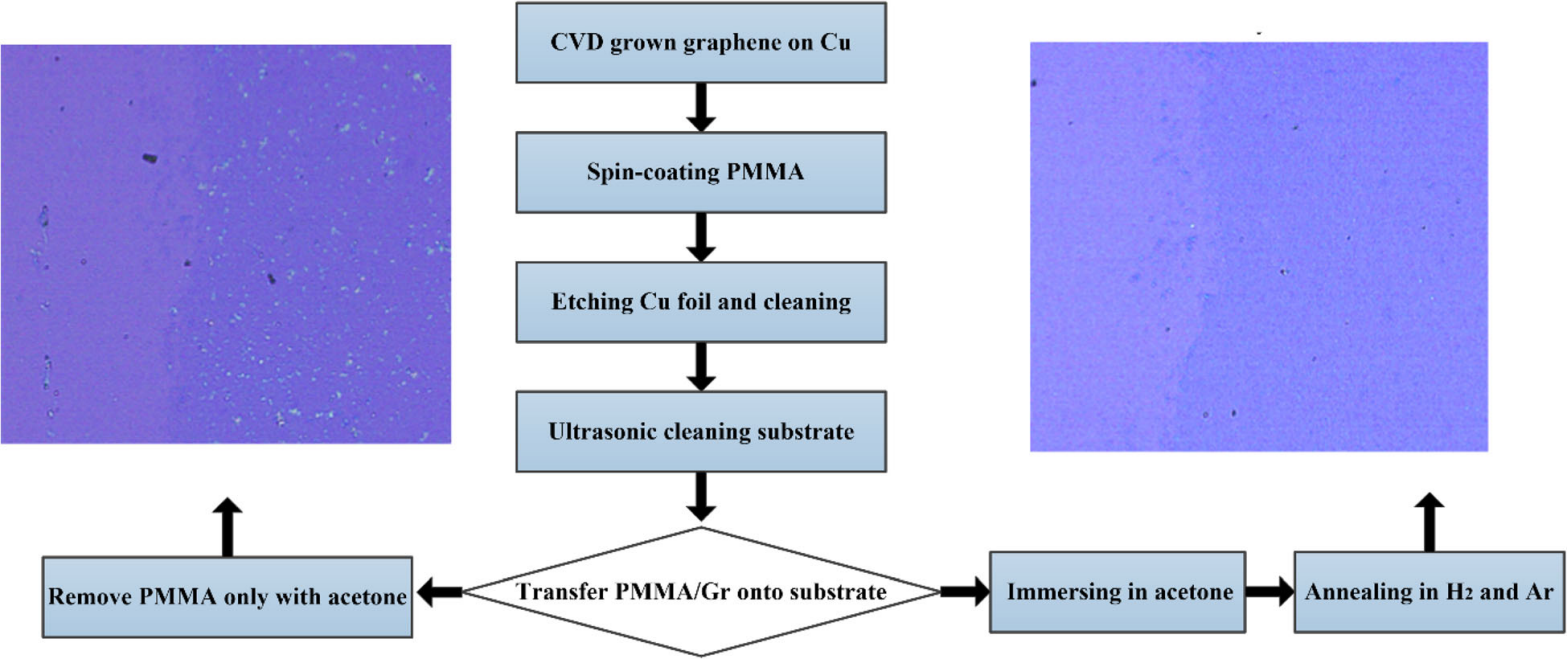
Processes for chemical transfer of graphene w/o annealing in reducing atmosphere. The insets are the optical photographs of graphene transferred on SiO_2_/Si substrate with (right) or without (left) annealing, respectively

**Fig. 2:**
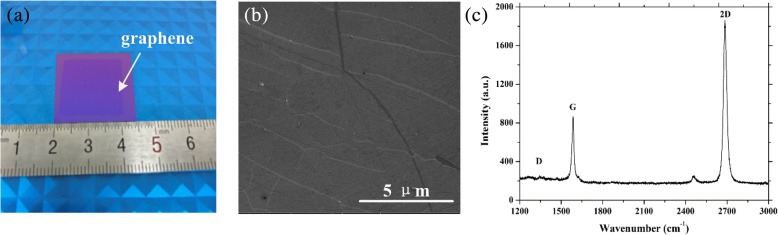
Optical photograph of a 2 × 2 cm^2^ graphene on SiO_2_/Si substrate (**a**). SEM image of the transferred graphene (**b**). Typical Raman spectrum showing the basic features of graphene (**c**)

However, we observed that the traditional wet transfer process could lead to cracks or wrinkles on the graphene surface with massive PMMA residue, which may greatly influence the electrical performance afterwards. As a result, we further utilized the ultrasound [23] to clean the SiO_2_/Si substrates with a post-annealing process based on the traditional wet transfer method, as shown in Fig. 1. Combining with 1-h ultrasonic treatment (power of 100 W and frequency of 50 Hz), both hydrophilicity and flatness of the substrate were enhanced, that a 2 cm × 2 cm graphene membrane could be continuously transferred to the substrate (Fig. 2a). In addition, we introduce a post-thermal annealing process [24, 25] to effectively remove the PMMA residue, with a mixing flow of Ar_2_ (100 sccm) and H_2_ (40 sccm) at 300 °C for 3 h. The details and discussion of optimization process are shown in Additional file 1.

Figure 2a clearly shows the optical photograph of the produced 2 cm × 2 cm graphene film on SiO_2_/Si substrate, indicating the excellent transparence of graphene. The graphene/SiO_2_ was characterized by field emission scanning electron microscope (Quanta 200 FEI), as is shown in Fig. 2b. The SEM image demonstrates that the graphene was continuous and uniformly transferred onto the substrate with few cracks or winkles. Moreover, Raman spectroscopy (514-nm laser excitation) is commonly used to evaluate the quality of the transferred graphene. Figure 2c shows the typical Raman spectrum of the graphene on SiO_2_/Si substrate. With unconspicuous D peak located at 1349 cm^−1^, the G and 2D peaks could be clearly observed at 1587 and 2685 cm^−1^ with a 2D/G ratio of 2.19. The low intensity of the D peak demonstrates that few additional defects were generated during the transfer process. The 2D peak is narrow with ratio I_G_/I_2D_ below 0.5, which indicates the basic features of single-layer graphene. The Raman spectrum results show high quality and continuity of the graphene with our optimized chemical transfer method.

### Fabrication of Graphene-Based Nanoscale Vacuum Channel Transistor

**Fig. 3:**
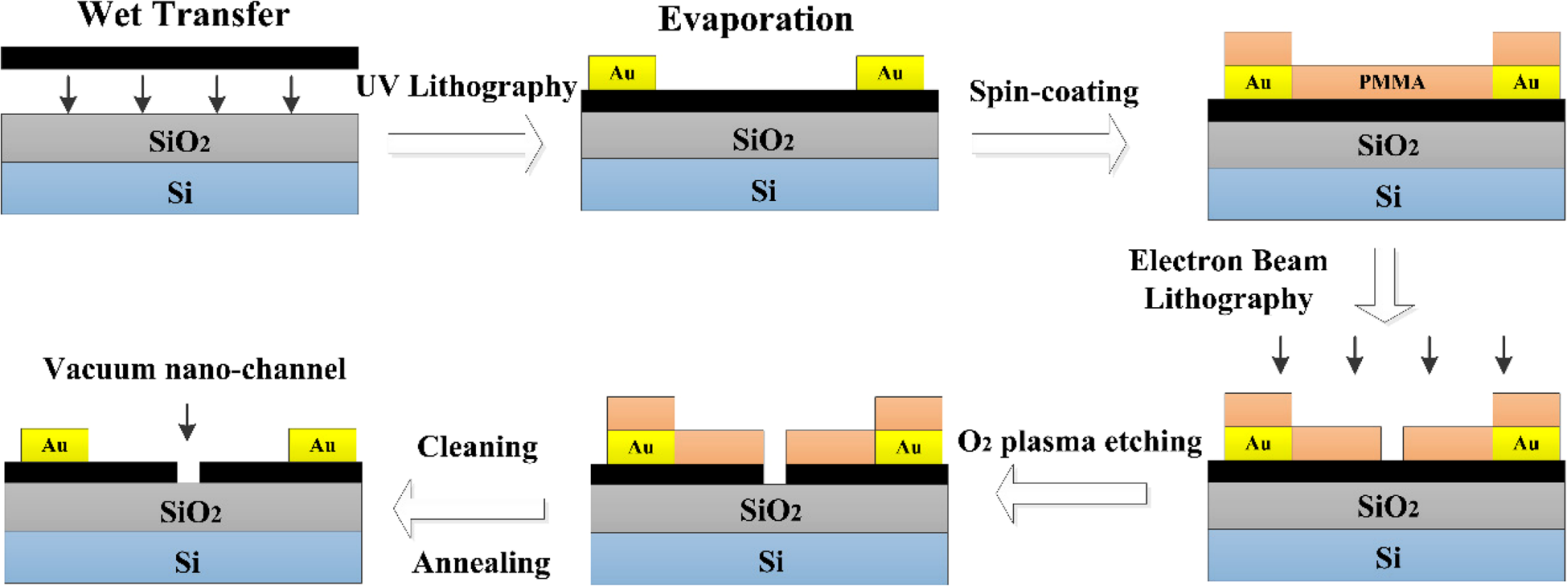
Schematic diagram of the fabrication process of the graphene-based nanoscale vacuum channel transistor

**Fig. 4:**
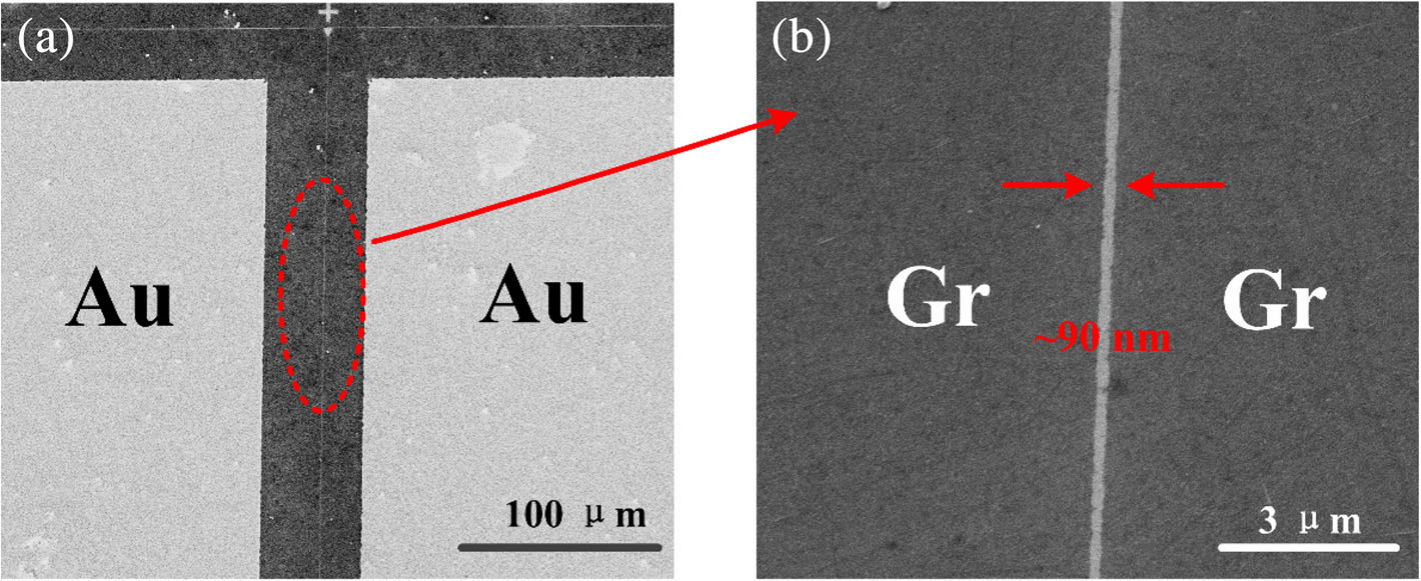
SEM image of graphene-based NVCT with Au contacts (**a**). A zoom-in of the ~ 90 nm vacuum channel (**b**)

Figure 3 illustrates the process of fabricating graphene-based nanoscale vacuum channel transistor. Firstly, the 100-nm SiO_2_ insulator was deposited by the PECVD (plasma enhanced chemical vapor deposition) method, with graphene chemical transferred onto the substrate subsequently. Gold contacts were deposited on graphene by electron beam evaporation (5 nm Cr and 80 nm Au) with a subsequent lift-off process. After PMMA spin-coated on the surface of graphene, the nano-vacuum channel was formed by standard EBL (Vistec, EBPG 5000plus ES) with a followed O_2_-plasma etching. The nanogaps were positioned to cut the graphene membrane into two halves. The samples were cleaned with acetone, isopropyl alcohol, and deionized water, respectively. Lastly, the samples were processed via 1 h of annealing at 300 °C with the flow of hydrogen (40 sccm) and argon (100 sccm). Figure 4a shows the SEM image of graphene-based NVCT, with Au contacts on both sides of graphene emitter and collector. And Fig. 4b demonstrates a zoom-in of the NVCT, showing approximately 90-nm-width vacuum channel that enables the electrons to ballistic transport through the nanogap.

## Results and Discussion

**Fig. 5:**
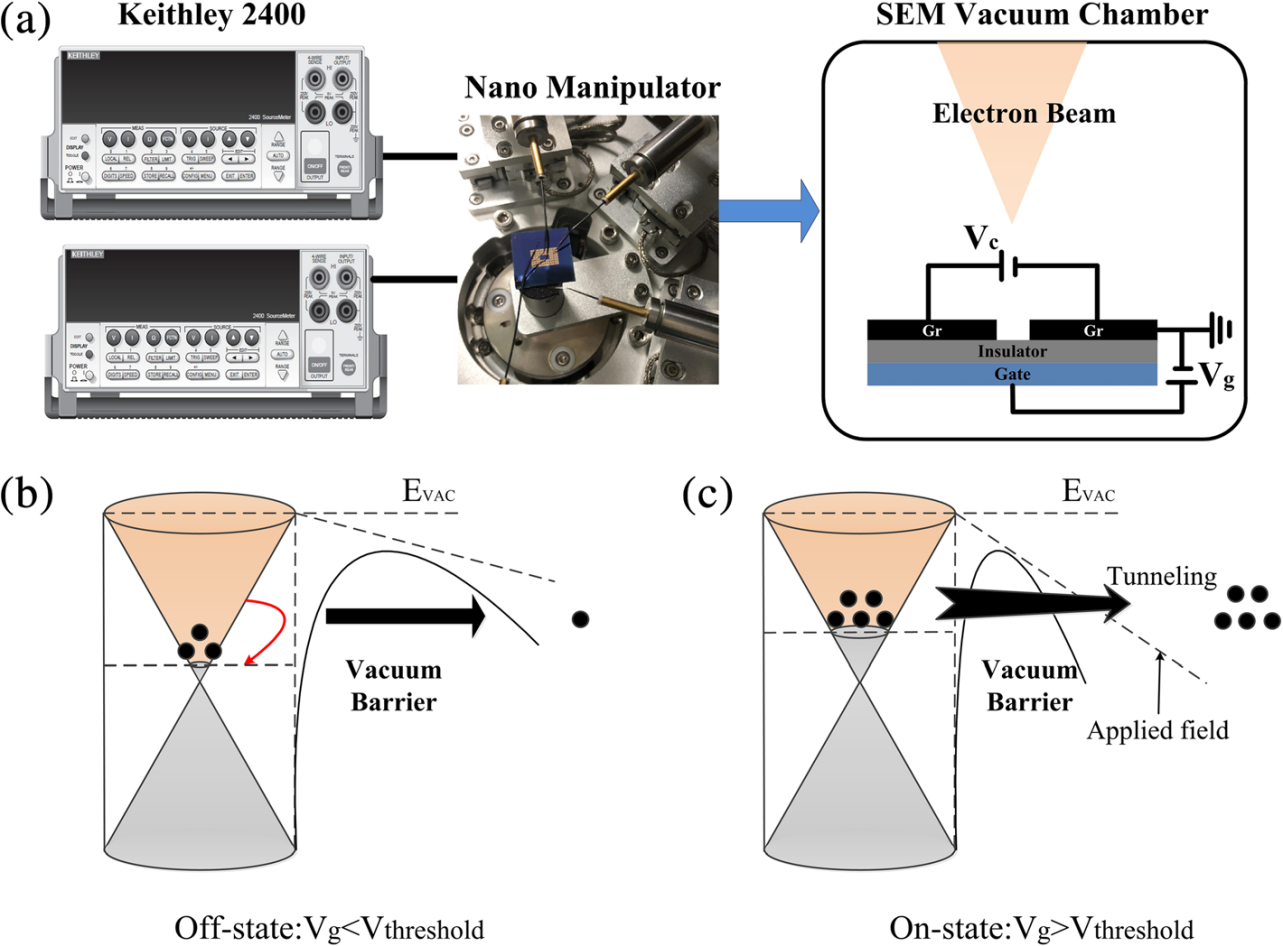
In-situ field emission measurement of the graphene-based vacuum nano-channel transistor (**a**). Band diagram of graphene-based NVCT at *V*_g_ < *V*_threshold_ and *V*_g_ > *V*_threshold_ (**b**, **c**)

To study the mechanism of electron transport through the vacuum nano-channel, the in-situ field emission measurements are performed with a nanomanipulator in the vacuum chamber of SEM (base pressure of ~ 10^−4^ Pa), as shown in Fig. 5a. The nanomanipulator system was developed for the real-time observation and measurement of field emission in vacuum environment, which could be considered as the probe station inside the SEM chamber and enable to locate or test the samples. Also, the in situ test method could reflect the electric properties of the graphene-based NVCT more objectively and serve the design of nanogap structure better. The nanomanipulator is equipped with cylinder-shaped tungsten microtips and connected to Keithely 2400 digital source measure unit. In order to avoid vacuum breakdown and damage of graphene, a current limit of 10 μA was imposed during the testing process. A bias was applied between the separated graphene films and increased manually at a voltage step of 0.1 V, that the electrons are emitted laterally from the graphene edges.

Figure 5b, c shows the band diagram of graphene-based NVCT at on- and off-states, respectively. Generally, the gate voltage applied at the back-gate could modulate the vacuum barrier between emitter and collector. When the gate voltage is less than the threshold voltage, the barrier is too broad to field tunneling for low-energy electrons. Also, the electrons might receive scattering by the impurities on the SiO_2_ surface and trapped to the drawbacks of the emitter. As the gate voltage increases beyond the threshold voltage, the width of the barrier is compressed accordingly. The electrons could overcome the narrowed barrier via the F-N tunneling, leading to the on-state of the NVCT. Moreover, the tunability of graphene energy band by gate voltage may be another contribution, as electrical conductivity of single-layer graphene can be modulated by gate voltage. As the gate voltage increases, the Fermi level E_F_ shift to the conduction band, thus, enhance the electron density of the graphene surface and improve the emission current.

**Fig. 6:**
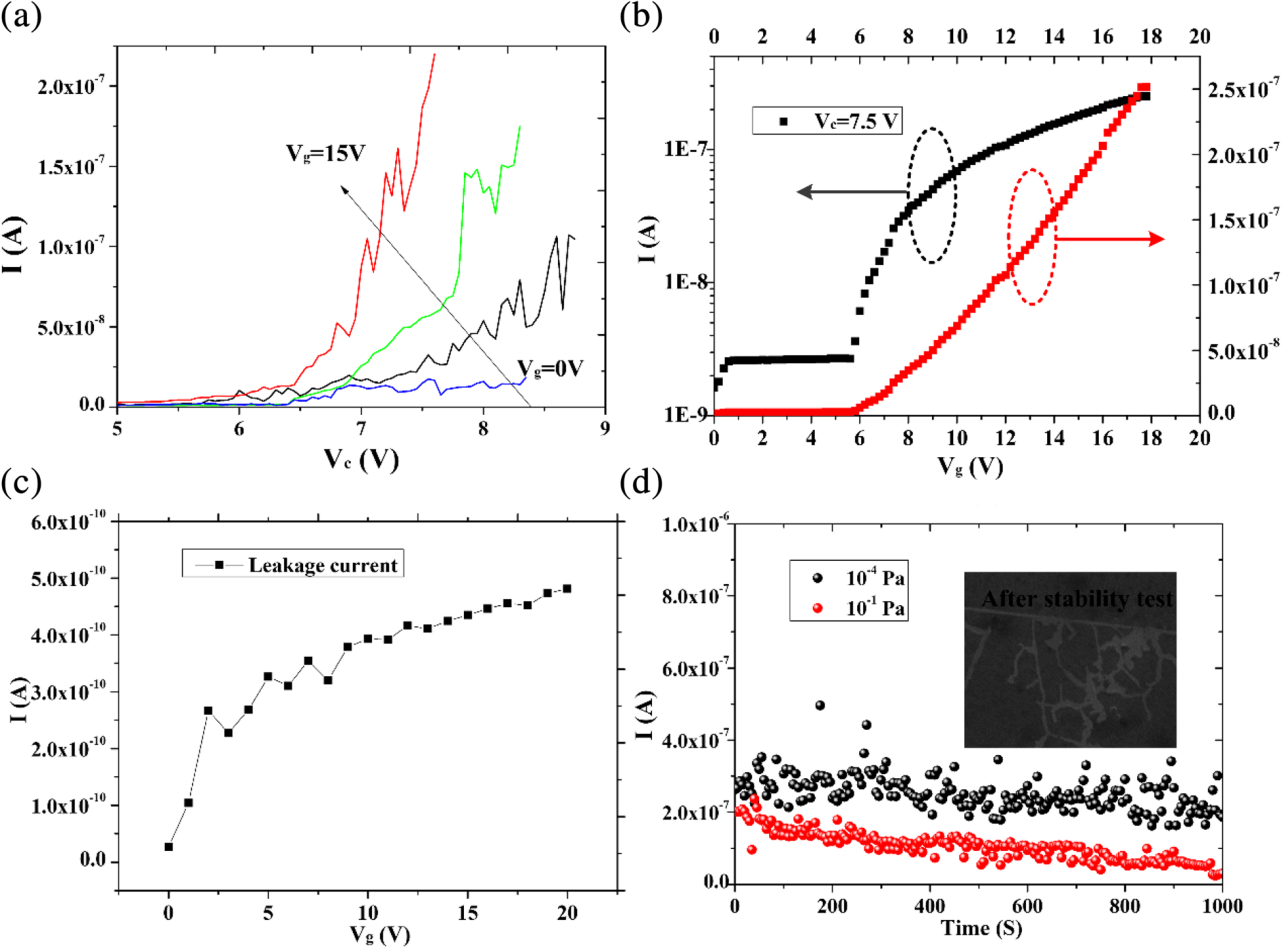
The output characteristics with V_g_ from 0 to 15 V (**a**). The transfer characteristics shows an on/off ratio exceeding 10^2^ (**b**). Leakage current of graphene-based NVCT (**c**). Stability test at different vacuum degrees (**d**). The inset shows the surface geometry changes after stable testing

To further explore the electric properties and extend the applications of graphene-based NVCT, the output (V_c_ vs. I_c_) and transfer (V_g_ vs. I_c_) characteristics are investigated, as is shown in Fig. 6a, b, respectively. Similar to the typical graphene-based field effect transistors (FETs), the graphene-based NVCT could be modulated in off-state or on-state by the gate voltage. Figure 6a illustrates the typical output characteristic with gate voltage *V*_g_ increasing from 0 to 15 V. It is noticed that no obvious electron emission *I*_c_ were measured when *V*_g_ is less than the threshold voltage, indicating that the NVCT is in the off-state. As the *V*_g_ increases and exceed the threshold voltage, the NVCT switches to the on-state that *I*_c_ exhibit an exponentially growth with collector voltage *V*_c_. The transfer characteristic with *V*_c_ = 7.5 V is shown in Fig. 6b in liner (red line) and exponential (black line) scale, respectively. We can see that the threshold voltage is about 6 V with a fixed collector voltage of 7.5 V, and *I*_c_ grows rapidly when *V*_g_ is larger than the threshold voltage. Also, the curves plotted in exponential scale (black line) exhibit an on/off ratio exceeding 10^2^, which is superior to the intrinsic graphene FETs due to the lack of bandgap. Wei et al. supposes that the electron emission properties are related to the surface topography of graphene or the distance from emitter to collector [12]. Thus, further narrowing of the nanogap width or modifying the structure may enable to enhance the on/off current ratio and electron emission.

To rule out the possibility of electron emission through the insulator, we also detect the leakage current during the measurement. Low and negligible leakage current (less than 0.5 nA) is observed, owing to the 100-nm thick SiO_2_ insulator. With a back-gate structure, however, the insulator plays a crucial role in the device. A thin insulator could enhance the modulation ability of back-gate while the insulator should be strong enough to avoid breakdown. As a result, optimizing the insulator material to decrease the thickness and improve the breakdown strength, e.g., utilizing Al_2_O_3_ or HfO_2_ as the high-k gate insulator [26–31], could indeed enhance the electric performance of the NVCT. Besides, the stability test of the NVCT at different vacuum degrees is shown in Fig. 6d with a fixed collector and gate voltage set as 7.5 V and 15 V, respectively. With the high thermal conductivity of graphene, the decrease of emission current induced by Joule heating is weakened, showing no obvious degradation and fluctuation at a vacuum degree of ~ 10^−4^ Pa. However, a slowly current reduction is observed in low vacuum (~ 10^−1^ Pa). The inset clearly exhibits the fracture and cracks on the graphene surface after stability test. It is supposed that the Joule heat aggregates at the graphene emitter and damages the surface morphology, leading to the emission current degradation in low vacuum [32, 33]. We hope that it could be solved in the further work, so that widens the application scope and occasion of the graphene-based NVCT.

To compare the performances of nanoscale vacuum channel transistors based on different types or materials, the channel width, operating voltage, working current, on/off ratio, gate current, and stability test are listed in Table 1. Obviously, the Si-based vacuum channel transistors (back-gate and gate-all-around) illustrate better performance than the graphene-based devices. By comparing the gate current, it can be seen that the energy consumption of our graphene-based NVCT are superior to the other devices. Meanwhile, the 90-nm-width vacuum channel could enable to scale down the size of vacuum devices and fulfill on-chip NVCT with multiple functionalities. However, the performances of on/off ratio or working current of our device are far behind with other structures and still need further improvement on the optimization of fabrication process and structure parameters. We hope that it could be presented in a future publication.

**Table 1:** Comparison of the performances of nanoscale vacuum channel transistors

Device Types	Channel width (nm)	Operating voltage (V)	Working current (nA)	On/off ratio	Gate current (nA)	Stability (*S*)
Vertical graphene-based vacuum transistor [12]	~ 300	< 10	10	10^6^	~ 10^6^	–
Planar back-gate vacuum channel transistor [9]	~ 150	< 20	4 × 10^4^	10^6^	–	–
Gate-all-around vacuum channel transistor [8]	~ 50	< 5	1000	10^3^	< 1	–
This paper	~ 90	< 20	200	10^2^	< 0.5	1000

## Conclusion

In conclusion, a graphene-based NVCT was successfully fabricated with standard CMOS process. We utilized the ultrasound to clean the SiO_2_/Si substrates with a post-annealing process based on the traditional wet transfer method that a 2 cm × 2 cm graphene membrane could be continuously transferred to the substrate. The electrical properties of NVCT were investigated. By modulating the gate voltage, the NVCT could be switched from off-state to on-state, exhibiting an on/off current ratio up to 10^2^ with low working voltages (< 20 V) and leakage current (< 0.5 nA). Further improvement of the graphene-based NVCT by structure optimization may pave the way for high speed, high reliability, and low cost applications for modern vacuum nanoelectronics.

## Additional File

Additional file 1: **Figure S1.** The schematic diagram and optical picture of the CVD system. **Figure S2.** The whole structure of the devices. Figure S3. Schematic diagrams of the carriers in solid-state device and the electrons in the vacuum nanogap. (DOCX 719 kb)

## Abbreviations

CVD: Chemical vapor deposition
EBL: Electron beam lithography
FET: Field effect transistor
FIB: Focused ion beam
IC: Integrated circuit
NVCT: Nanoscale vacuum channel transistor
PECVD: Plasma-enhanced chemical vapor deposition
PMMA: Polymethyl methacrylate
SEM: Scanning electron microscope
