# Supplementary material for: Graphene-Based Nanoscale Vacuum Channel Transistor
Source: Nanoscale Res Lett. 2018 Oct 4;13:311. doi: 10.1186/s11671-018-2736-6 (PMC6172161; doi:10.1186/s11671-018-2736-6)
Supplement: Supplementary file 1 — Figure S1. The schematic diagram and optical picture of the CVD system. Figure S2. The whole structure of the devices. Figure S3. Schematic diagrams of the carriers in solid-state device and the electrons in the vacuum nanogap. (DOCX 719 kb) [file 11671_2018_2736_MOESM1_ESM.docx]

1. **Fabrication of graphene**


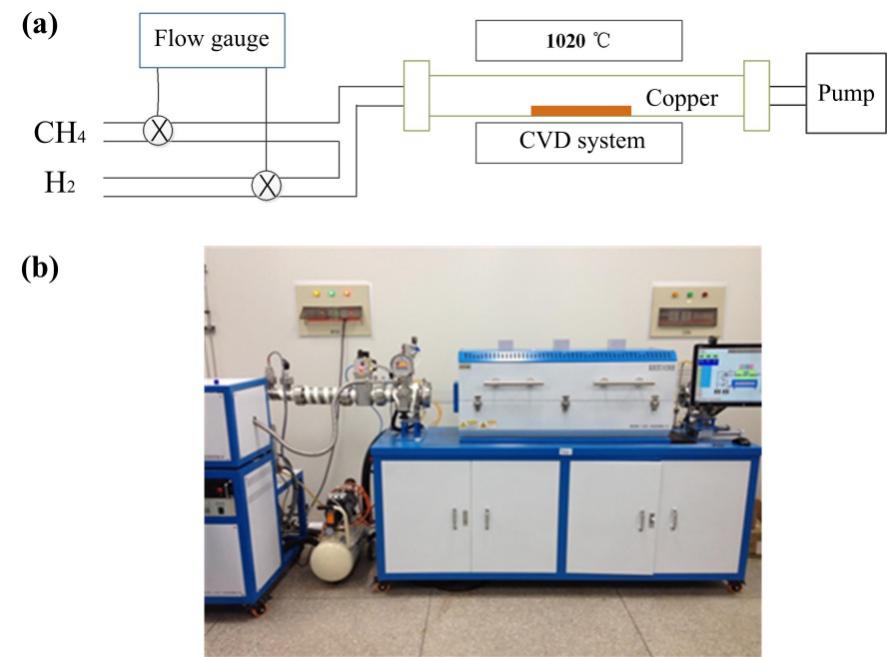


Fig. s1 The schematic diagram and optical picture of the CVD system

In this report, large-scale graphene was directly grown on the Cu foil by thermal chemical vapor deposition (CVD) at 1020 °C with CH_4_ (20 sccm) and H_2_ (40 sccm), as we reported previously[[1](#_ENREF_1)].

1. **Optimization of graphene surface**

We observed that the traditional wet transfer process could lead to cracks or winkles on the graphene surface with massive PMMA residue, which may greatly influence the electrical performance afterwards. One possible cause is that graphene could hardly contact with the hydrophobic substrate tightly, thus be torn or damaged in the subsequent transfer process. To a certain extent, the surface properties of substrate may affect the quality of transferred films. During the transfer process, few water molecules would remain between the substrate and graphene. After drying and evaporating the water molecules, the surface tension would induce the contact of graphene and substrate. With a hydrophobic substrate, the distribution of water molecules would be extremely irregular. And the drying process may produce bubbles at the interfaces and result in poor interfacial contact, leading to cracks and wrinkles on the graphene surface[[2](#_ENREF_2), [3](#_ENREF_3)]. Meanwhile, the ultrasonic treatment could remove the hydrophobic amorphous materials adhered to the substrate, improving the substrate hydrophilicity so that the graphene could fully contact with the substrates[[4](#_ENREF_4)].

On the other hand, it is known that PMMA layer plays a crucial and irreplaceable role in the wet transfer process. It can effectively support the graphene films and accurately target the substrate, avoiding the extensive tearing of graphene. Nevertheless, amounts of PMMA would remain on the graphene surface by removing PMMA with acetone only, which would cause severe P-type doping. These adhesive residue will greatly affect the transferred graphene, hinder further exploration of its inherent electrical properties and become one of the key factors limiting the device performance. Previous studies have demonstrated that the thermal annealing process could be considered as an effective method[[5](#_ENREF_5), [6](#_ENREF_6)]. After long-time and high-temperature burning in the reducing atmosphere, the internal molecular chains of the PMMA can be unwrapped, thereby reducing their adhesion and facilitating detachment.

As a result, we further utilized the ultrasound to clean the SiO_2_/Si substrates with a post-annealing process based on the traditional wet transfer method, as is shown in Fig .1. Combing with one-hour ultrasonic treatment (power of 100W and frequency of 50Hz), both hydrophilicity and flatness of the substrate were enhanced, that a 2cm×2cm graphene membrane could be continuously transferred to the substrate (Fig. 2(a)). In addition, we introduce a post-thermal annealing process to effectively remove the PMMA residue, with a mixing flow of Ar_2_ (100 sccm) and H_2_ (40 sccm) at 300°C for 3 hours.


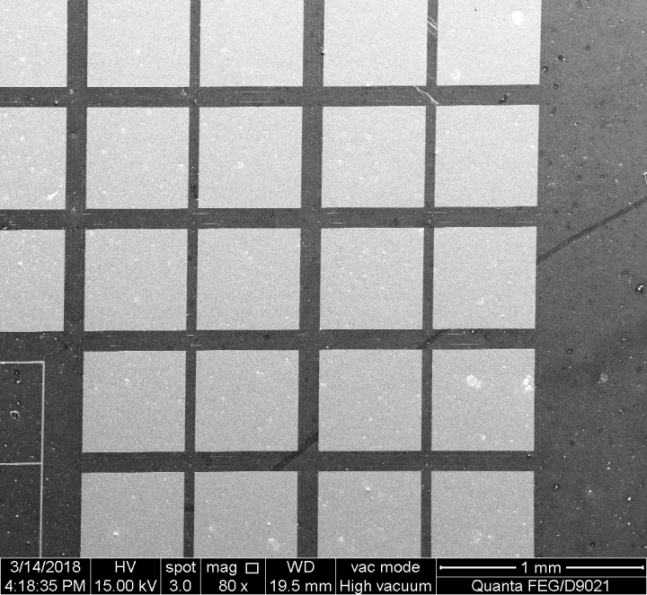


Fig. s2 the whole structure of the devices

Fig. s2 shows the whole structure of the NVCT devices. The nanogap channel from emitter to collector is ~90 nm. And the length in the perpendicular direction is about 500 μm. Besides, the devices are separated from each other by the EBL and a following O_2_ plasma etching, which could be seen in Fig. 1(b).


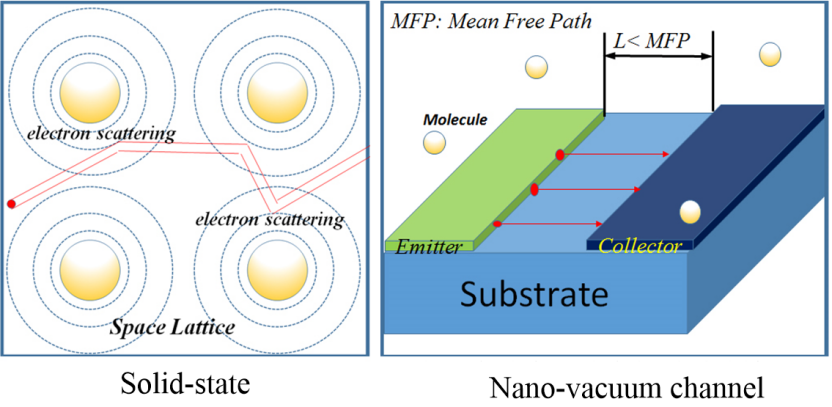


Fig S3 Schematic diagrams of the carriers in solid-state device and the electrons in the vacuum nanogap

Figure S3 shows the carriers transport in the channel of the field-effect transistor (left) while the electrons travel in a nanoscale vacuum channel (right). For conventional FETs, the carriers may collide with the optical and acoustic phonons during the transport. Besides, intrinsic graphene-based FETs were found to have an On-Off current ratio less than 10 due to the lack of a bandgap, that are not suitable for modern integrated logic circuits.

As a result, graphene-based NVCTs may have at least two advantages compared to the charge carriers transport in graphene channels or other solid-state mediums. To start with, charge carriers transport freely in vacuum without any scattering, as is shown in Figure r1. On the other hand, by applying a vacuum tunneling barrier, graphene-based NVCTs could output sufficient On-Off current ratio. Moreover, the vacuum nano-devices could be compatible with standard silicon process and combine the advantages of ballistic transport with miniaturization and integration. More importantly, the NVCT is proved to retain the advantages of the traditional vacuum tubes that operate normally in the extreme conditions, like exposure of ionizing radiation or high temperature. The development of manufacturing technology can open up enormous opportunities for creating nanoscale vacuum channel, which might be compatible with modern integrated circuit (IC).

1. Xu J, Wang QL, Tao Z, Qi ZY, Zhai YS, Wu SQ, Zhang XB, Lei W (2016) *Field Emission of Wet Transferred Suspended Graphene Fabricated on Interdigitated Electrodes. Acs Applied Materials & Interfaces 8* (5): 3295-3300.

2. Li XS, Zhu YW, Cai WW, Borysiak M, Han BY, Chen D, Piner RD, Colombo L, Ruoff RS (2009) *Transfer of Large-Area Graphene Films for High-Performance Transparent Conductive Electrodes. Nano Lett. 9* (12): 4359-4363.

3. Liang XL, Sperling BA, Calizo I, Cheng GJ, Hacker CA, Zhang Q, Obeng Y, Yan K, Peng HL, Li QL, Zhu XX, Yuan H, Walker ARH, Liu ZF, Peng LM, Richter CA (2011) *Toward Clean and Crackless Transfer of Graphene. ACS Nano 5* (11): 9144-9153.

4. Yuan LY, Tao YT, Chen J, Dai JJ, Song T, Ruan MY, Ma ZW, Gong L, Liu K, Zhang XH, Hu XJ, Zhou J, Wang ZL (2011) *Carbon Nanoparticles on Carbon Fabric for Flexible and High-Performance Field Emitters. Advanced Functional Materials 21* (11): 2150-2154.

5. Pirkle A, Chan J, Venugopal A, Hinojos D, Magnuson CW, Mcdonnell S, Colombo L, Vogel EM, Ruoff RS, Wallace RM (2011) *The effect of chemical residues on the physical and electrical properties of chemical vapor deposited graphene transferred to SiO2. Appl. Phys. Lett. 99* (12).

6. Xu J, Wang QL, Tao Z, Qi ZY, Zhai YS, Lei W, Zhang XB (2016) *Enhanced electron emission of directly transferred few-layer graphene decorated with gold nanoparticles. Rsc Advances 6* (81): 78170-78175.
